# Integrating HIV and Mental Health Services for Black Gay, Bisexual, and Other Men Who Have Sex with Men Living with HIV: Findings from the STYLE 2.0 Intervention

**DOI:** 10.1089/apc.2022.0141

**Published:** 2022-09-30

**Authors:** Sara H. LeGrand, Dirk A. Davis, Heather E. Parnell, Elizabeth J. Trefney, Brian Goings, Ta'Jalik Morgan

**Affiliations:** Center for Health Policy and Inequalities Research, Duke Global Health Institute, Duke University, Durham, North Carolina, USA.

**Keywords:** Black men who have sex with men, mental health, health care navigation, HIV

## Abstract

Black gay, bisexual, and other men who have sex with men (BMSM) in the US South are disproportionately impacted by HIV. We adapted Project Strength Through Youth Livin’ Empowered (STYLE) to create STYLE 2.0 to assist young BMSM link and remain engaged in HIV care. The multi-component intervention included (1) health care navigators to facilitate linkage and engagement activities, (2) motivational interviewing by a behavioral health provider, and (3) a mobile app to reduce stigma and social isolation. We enrolled 66 BMSM from North and South Carolina in the 12-month intervention and analyzed longitudinal data to assess service utilization, dose, and delivery characteristics while also examining changes in HIV care continuum outcomes. We examined associations between intervention characteristics and HIV care continuum outcomes using logistic regression. We found that all HIV outcomes improved from baseline to 12-month follow-up, including receipt of HIV care (78.8–84.9%), retention in HIV care (75.9–87.7%), being prescribed antiretroviral therapy (ART) (96.8–98.5%), and achieving viral suppression (82.3–90.8%), although none were statistically significant. In multi-variable analyses, participants with more encounters categorized as food bank were more likely to report being prescribed ART [odds ratio (OR): 41.65; 95% confidence interval (CI): 2.72–637.74]. Clients with more referral to care encounters were less likely to have been prescribed ART (OR: 0.02; 95% CI: <0.001–0.42) and be virally suppressed (OR: 0.39; 95% CI: 0.18–0.84). Findings suggest that an integrated approach to HIV and behavioral health services may help BMSM living with HIV overcome structural and social barriers to HIV care.

## Introduction

Since the start of the HIV epidemic in the United States, Black gay, bisexual, and other men who have sex with men (BMSM) have experienced HIV-related disparities such as higher HIV incidence and prevalence and poorer outcomes along the HIV Care Continuum, including lower rates of retention in HIV care, antiretroviral therapy (ART) uptake, and viral suppression.^1–6^ Black Americans are 8.1 times more likely to be diagnosed with HIV than White Americans and account for 43% of HIV diagnoses, despite representing only 12% of the total US population.^7,8^ In addition, rates of HIV diagnosis are higher among adolescents and young adults, particularly young BMSM.^9^ During 2008–2016, young BMSM accounted for 49% of all HIV diagnoses among men who have sex with men (MSM) aged 13–29 years.^10^

Owing largely to underlying structural and societal factors, the US South has the highest levels of HIV diagnoses and HIV deaths of all US regions.^11^ Although diagnoses among MSM leveled out in other regions of the country for many years, they continued to rise significantly in the Deep South (Alabama, Georgia, Florida, Louisiana, Mississippi, North Carolina, South Carolina, Tennessee, and Texas).^12–15^ In the Deep South, BMSM represent ∼33% of all HIV diagnoses and BMSM, including young BMSM, have poorer HIV care outcomes compared with White MSM across the HIV Care Continuum.^16–18^ According to the Centers for Disease Control and Prevention (CDC), BMSM in the Southern United States face a variety of barriers to testing, linkage, and retention in HIV prevention and treatment services, including racism, lower educational levels, stigma, income inequality, and lack of access to health care are.^19^ And for BMSM living in rural areas of the US southeast often have to travel more than 80 km to receive HIV care.^19^

HIV disparities experienced by BMSM are not the result of greater participation in HIV risk behaviors compared with other MSM,^3,20^ but rather due to factors such as stigma and discrimination associated with minoritized intersecting identities (e.g., race, ethnicity, sexual orientation, and HIV status), a lack of social support, poverty, medical mistrust, and underfunded HIV prevention efforts tailored to BMSM.^21–26^ In addition, young BMSM experience gaps in the HIV care continuum as a result of mental health issues, including depression, anxiety, substance use, and trauma-related disorders.^27,28^ These mental health issues, such as depression and post-traumatic stress disorder (PTSD), are more prevalent among people living with HIV and those with a greater risk of contracting HIV, compared with the general population.^29,30^

Lower rates of mental health service utilization combined with elevated stress, hopelessness, and low self-esteem exacerbated by these behavioral health conditions lead to negative outcomes along the HIV care continuum.^28,29,31–33^ Specifically, depressive symptoms are associated with decreased HIV symptom management, medication adherence, and viral suppression.^28,31,34,35^ PTSD also increases the likelihood of nonadherence and overall worse HIV health outcomes.^32,36^ By integrating behavioral health and HIV care services, providers may help to address treatment barriers and improve care continuum outcomes among BMSM living with HIV.^28,35^

Patient navigation is one intervention strategy that may meet the complex needs of BMSM living with HIV by reducing structural barriers, such as the need for multiple referrals to outside organizations, as well as financial and individual-level barriers, including medical distrust and the knowledge needed to navigate a disconnected health care system.^37,38^ In this patient-centered role, navigators work 1:1 with clients to assist with key tasks, including appointment scheduling and reminders, accompanying clients to appointments, sharing HIV and general health information, or providing referrals.^37,39^ Successful health care navigation is correlated with improved linkage and retention in care and viral suppression.^37,40^ To improve navigator acceptance, health care navigators (HCN) and their clients should share similar racial, cultural, and/or sexual identities.^41–44^

To address these needs, the Health Resources and Services Administration Special Projects of National Significance funded the initiative *Implementation of Evidence-Informed Behavioral Health Models to Improve HIV Health Outcomes for Black Men who have Sex With Men* in 2018. This initiative funded the adaptation, implementation, and evaluation of evidenced-informed models of care to help engage BMSM in HIV medical care and other behavioral health and supportive services. Project Strength Through Youth Livin’ Empowered (STYLE) was one of the proposed models of care. It utilized an innovative model of care designed to engage and retain young Black and Latino MSM living with HIV into HIV care.

The current project adapted STYLE to create STYLE 2.0 for young BMSM living in the Triangle region of North Carolina (Durham, Orange, and Wake counties) and the Columbia, South Carolina area. The aims of this study were to (1) adapt STYLE to STYLE 2.0 using a community-engaged approach, (2) assess service utilization, dose, and delivery characteristics, (3) examine changes in HIV care continuum outcomes over the intervention period, and (4) explore associations between service utilization, dose, and delivery characteristics and HIV care continuum outcomes.

## Methods

### Adaptation of original STYLE intervention

Project STYLE was an innovative model of care designed to engage and retain Black and Latino young men who have sex with men (YMSM) living with HIV, ages 17–24 years into HIV care.^45^ Enhancements and adaptations to STYLE for STYLE 2.0 that focused only on young Black men who have sex with men (YBMSM) living with HIV ages 18–35 years included (1) HCN to facilitate all linkage and engagement activities, (2) a motivational interviewing (MI) intervention delivered using videoconferencing by a Behavioral Health Provider, and (3) a mobile application (mobile app) to facilitate and provide additional resources, connections to HCN, online support groups and peer-to-peer sharing to reduce stigma and social isolation. Table 1 shows specific components updated and adapted for STYLE 2.0 from the original STYLE intervention.

**Table 1.** Original STYLE Components and STYLE 2.0 Components

Original STYLE component	STYLE 2.0 components
A social marketing campaign developed with the input of a YAB and focus groups.	YAB assisted with social marketing campaign adaptations as well as overall program implementation, evaluation, and dissemination.
	Full access to original STYLE social marketing materials that were reviewed and adapted as needed based on YAB input.
	In-person social marketing included outreach and distribution of materials to locations frequented by YBMSM.
	Recognition of the original STYLE brand to foster interest and trust among YBMSM.
	YAB convened to provide input on the imagery and content of social media recruitment ads and appropriate sites for engaging YBMSM.
	Social marketing resources dedicated to advertising the project on social networking sites popular among YBMSM such as Facebook, Instagram, and Twitter.
Intensified outreach to young Black and Latino MSM youth-serving venues and to increase provision of HIV testing services on college campuses, and within the broader community utilizing both venue-based and social and sexual network testing approaches. Strong relationships with NC Disease Intervention Specialists/Bridge Counselors assisted with identification of young Black and Latino MSM who had fallen out of care.	Outreach to venues frequented by YBMSM to identify individuals eligible for intervention participation.
	Continued to rely on close relationships with Triangle area community organizations to identify YBMSM who have never engaged in HIV care or have fallen out of care.
	Monthly meetings with clinic medical staff and SS staff to identify YBMSM at risk of falling out of HIV care.
	STYLE 2.0 app, developed through HealthMPowerment, provided information about the importance of linkage and regular engagement in HIV care.
	Program participants encouraged to share this information with individuals in their social and sexual networks to facilitate enrollment of other YBMSM.
	STYLE 2.0 app included a feature allowing out of care YBMSM to directly message HCN to facilitate engagement/re-engagement.
A tightly linked *medical–social support network* for youth newly diagnosed with HIV or re-engaging in care that included an infectious disease board-certified physician who oversaw the provision of care to all patients. Research team available to participants by phone and text messaging to assist with appointment scheduling or to answer medical questions.	Infectious disease physicians received warm handoffs from HCN and provided culturally competent care for YBMSM.
	HCN available by phone and text messaging to assist with scheduling appointments.
	HCN scheduled and attended clinic appointments with participants.
	HCN provided the physician with a summary of participant needs and barriers to care before appointments.
	The HCN and BHP available to assist with scheduling appointments or answering questions through a messaging portal embedded in the STYLE 2.0 app. They responded to all queries within 48 h.
Provided participants with ancillary social SS, including case management, mental health services, and weekly in-person support groups through a partnership with a local AIDS Service Organization.	HCN gave warm handoffs versus simple referrals to behavioral health services, case management services, and other services to reduce barriers linkage and regular engagement in HIV care.
	The BHP provided a four-session motivational interviewing intervention through videoconferencing through STYLE 2.0 app for those who screened positive for mental health or substance abuse issues or referred by clinic staff.
	The HCN held weekly virtual support group meetings through videoconferencing.

BHP, behavioral health provider; HCN, Health Care Navigators; MSM, men who have sex with men; SS, support services; STYLE, Strength Through Youth Livin’ Empowered; YAB, Young Adult Advisory Board; YBMSM, young Black men who have sex with men.

Adaptation of the STYLE 2.0 intervention occurred during the first 6 months of the project funding, August 2018 through January 2019. We chose to expand the age range for recruitment purposes to ensure we were able to enroll sufficient participants into the study, and in line with other studies who define “younger” between 18 and 35 years.^46^ All adaptations were informed by extensive guidance from a Young adult Advisory Board (YAB) of BMSM. The YAB consisted of 4–8 YBMSM living with HIV, the number fluctuated throughout the project as some YAB members moved away or were no longer available to participate, from the intervention regions that met monthly throughout STYLE 2.0 intervention development.

The YAB was convened as a new group specifically for STYLE 2.0. Potential YAB participants were identified through clinic staff, community members, and word of mouth. Although the YAB did not develop intervention activities, they were able to offer feedback and opinions on implementation of activities such as recruitment approaches and timing and language around warm hand offs to the behavioral health provider. The YAB also worked with STYLE 2.0 staff to review all of the original STYLE materials (e.g., logos and advertising materials) to determine which materials should be used or modified for use in STYLE 2.0. The YAB continued to meet regularly with the project team throughout the project to provide input on intervention implementation, evaluation, and dissemination. YAB members were reimbursed each meeting attended through an e-gift card for their time.

### Intervention description

STYLE 2.0 participants were able to choose their level of participation in the components of the intervention. All participants were assigned one of two STYLE 2.0 HCN and completed enrollment and assessment activities. HCN were located in the Durham, NC region and provided services to all STYLE 2.0 participants through phone or virtually. Navigators were chosen due to their involvement in Black lesbian, gay, bisexual, transgender, and questioning (LGBTQ) and HIV-centered communities and provided culturally competent services.

After enrollment HCN shared the various STYLE 2.0 components with participants, including HCN one-on-one sessions, behavioral health services, virtual support group, and the STYLE 2.0 app. Participants were then able to determine their level of participation, for example, support groups and app engagement were not mandatory, but available for those who chose to participate. Although HCN one-on-one sessions were not mandatory, they were highly encouraged. Navigators used a CDC-adapted program, Choosing Life: Empowerment, Actions, Results (CLEAR),^47^ which they used to assist participants in creating skills to help them make healthy life choices.

Participants met with the health care navigator to determine which key skills were the most important work on, and then had the option to meet with them every 1–2 weeks. Sessions included “Creating a Vision for the Future,” “Communication,” “Problem Solving,” and “Adherence.” The navigator also linked participants to supportive services (e.g., housing, education, and employment) and additional clinical and behavioral health care, as needed. The intervention also included a mobile app developed specifically for STYLE 2.0 through adaptations of the healthMpowerment app,^48^ a mobile app that contained community-building and educational features and was utilized in previous studies with STYLE 2.0 study team members.

In addition, STYLE 2.0 contained several behavioral health components, including the use of the Substance Abuse and Mental Illness Screener (SAMISS)^49^ at intake and 6 months. HCN were trained in administering and scoring the screener that screens for substance use and mental illness. If a participant screened positive on the screener they were then referred to the STYLE 2.0 behavioral health provider for MI sessions. For participants who screened positive for behavioral health services, navigators provided a warm handoff to the behavioral health provider who facilitated four virtual MI sessions. During these sessions, the provider helped the participant find the inner confidence and motivation necessary to make specific positive life changes.

General goals included increasing readiness to enter or regularly engage in HIV care and identifying and addressing the key barriers that prevent such engagement. In addition to one-on-one behavioral health, STYLE 2.0 also offered a virtual support group for participants throughout the intervention period. This weekly support group, facilitated by HCN, provided a space for participants to build community, offer one another support, as well as had hear educational presentations.

**FIG. 1.**
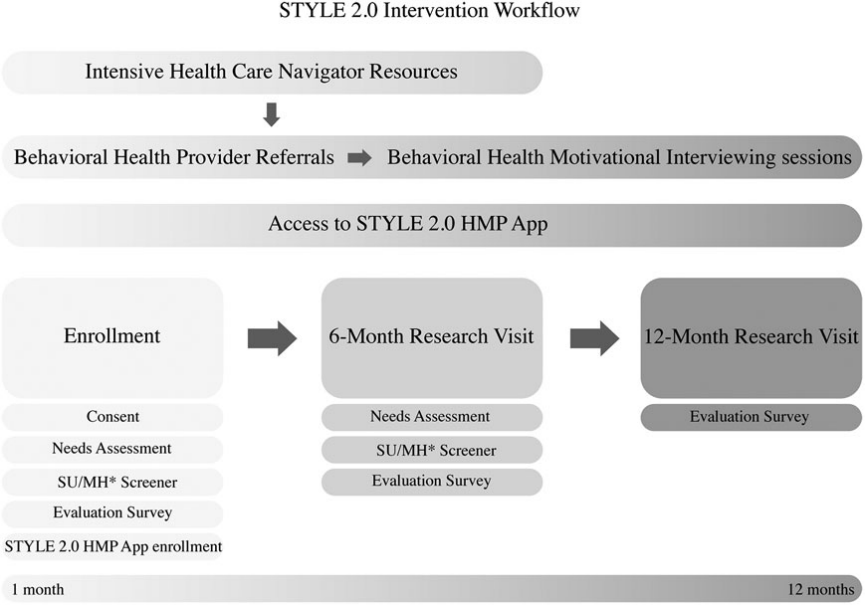
STYLE 2.0 intervention workflow. *Substance use/mental health screener. STYLE, Strength Through Youth Livin’ Empowered.

STYLE 2.0 intervention period lasted 12 months from the date of enrollment. The first 6 months included intense work with the navigators followed by a 6-month transition period where navigators were available as needed, but no prescribed outreach or sessions occurred (Fig. 1).

### Participant recruitment and enrollment

Participants were eligible for STYLE 2.0 if they were 18–35 years old at the time of enrollment, identifying as Black or African American, cisgender male, and residing in the study area in NC or SC. Participants were either newly diagnosed with HIV, new to HIV care, fallen out of care, at risk of falling out of care, or not virally suppressed. STYLE 2.0 partnered with several infectious disease clinics within the intervention area, including Duke University, University of North Carolina—Chapel Hill, Lincoln Community Health Center Early Intervention Clinic, University of South Carolina—Prisma Health, and Wake County Human Services. STYLE 2.0 utilized several recruitment methods, these included in-person recruitment from medical clinics, referrals from current participants and community members, as well as virtual recruitment methods from social media posts.

Medical care providers, nurses, social workers, and other clinic staff were trained in referring potential participants to one of two navigators or to the online pre-screener for screening. In addition, potential participants were referred through word of mouth from current STYLE 2.0 participants or other community members. Flyers and palm cards were placed at clinics that referred potential participants to the online screener for eligibility screening. Once a participant completed the screener, if eligible, participants shared their contact information and a navigator contacted them. Informed consent was obtained for all participants upon enrollment. STYLE 2.0 began recruiting for participants in September 2019 and our first participant enrolled in November 2019 and continued through December 31, 2020. Out of the 128 potential participants who were referred, 66 enrolled in STYLE 2.0. Ethical approval was granted by the Institutional Review Board at Duke University.

### Data collection

Participants completed self-administered surveys through Qualtrics software^50^ at three time points, including baseline, 6 months, and 12 months throughout the evaluation period that ended January 31, 2021. The survey included topics of mental health, substance use, sexual behavior, medication use, and use of health care services. The survey took ∼45 min to complete online and participants were compensated with a $50 e-gift card for baseline and $35 e-gift card for each follow-up survey.

In addition, medical chart abstractions were conducted at baseline, 6 months, and 12 months to collect data related to HIV medical care appointments, behavioral health and support services screening, referrals, and appointments, and laboratory and medication data. Intervention dosage data were collected through Qualtrics by navigators and behavioral health providers. All interactions with participants were documented in a tracking database within 24 h of participant interaction and included information on provider, length of interaction, type of interaction, and other detailed information.

### Measures

#### Dependent variables

##### Linkage to medical care

Newly diagnosed clients, as determined by their clinic to have been diagnosed within the past 12 months, who attended a routine HIV medical care visit within 3 months of HIV diagnosis (only measured at baseline); and newly diagnosed clients who attended a routine HIV medical visit within the past 3 months (measured at baseline, 6 months, and 12 months).

##### Receipt of HIV medical care

Clients who had two or more routine HIV medical care visits in the past year.

##### Retention in HIV medical care

Clients who had at least one routine HIV medical care visit in the past 12 months, with a second visit at least 90 days after.

##### Prescribed ART

Clients prescribed ART in the past 12 months.

##### Viral suppression

Clients with a HIV viral load <200 copies/mL at last HIV viral load test in the past 12 months.

##### Behavioral health

We assessed a variety of behavioral health variables all in the previous 6 months, including clients screened for behavioral health care needs, clients who screened positive for behavioral health care needs, clients with positive behavioral health needs who were referred to behavioral health care, clients with positive behavioral health needs who received behavioral health care, and clients with positive behavioral health needs who received four or more behavioral health visits.

##### Support services

We assess various social support variables all in the previous 6 months, including clients screened for support services, clients screened positive for support services care, clients with positive support services needs referred to support services, and clients with positive support service needs who received support service care. These data were obtained through clinical chart abstraction and represent support services provided by clinic staff, including medication program enrollment, financial assistance, and other types of support services.

#### Independent variables

To measure engagement with the intervention, we created independent variables using monitoring data documented by intervention staff over the 12-month intervention period.

##### Frequency of encounters

A count of the number of documented encounters of any type between the participant and intervention providers, this included both STYLE 2.0 HCN and the STYLE 2.0 behavioral health provider.

##### Encounter duration

Encounters categorized by duration in minutes: 0–30 min (reference), 31–60 min, and ≥60 min.

##### Encounter service category

A count of each type of encounter categorized by the intervention provider. These categories were created by the Ryan White HIV/AIDS Program to be used across intervention sites. Examples of each encounter service category are as follows:

- Psychosocial (reference)—individual check-ins by HCN; virtual support groups
- Medical case management—intake assessments; accompaniment to clinic appointments
- Nonmedical case management—referral to support services not listed (i.e., excluding food and housing)
- Mental health—MI sessions; ongoing individual counseling with Behavioral Health provider
- Food bank/meal delivery—connection to community-based organizations that provide food; getting on delivery lists for community food banks
- Health education/risk reduction—CLEAR sessions; health education not related to medical case management
- Housing—connecting participants to rent assistance services and COVID-19-related housing support
- Medical transport—providing transport to appointments; coordinating travel, including the use of medical Lyft
- Referral for care—referrals to health care and supportive services (i.e., behavioral health provider, medical providers, and insurance providers).

##### Contact type

A count of each type or mode of contact documented by the intervention provider: electronic health record, e-mail, group by phone, individual by phone (reference), mobile app, text, virtual support group, virtual visit (i.e., web chat). For multi-variable analyses, the virtual support group and virtual visit contact types were combined into one (virtual visit).

##### Provider type

Number of encounters provided by a health care navigator or behavioral health therapist.

### Data analysis

We used descriptive statistics to assess baseline characteristics of our sample (Table 1), participation in intervention components (Table 2), and engagement with the study-specific mobile app (Table 3). We used Fisher's exact test to assess if proportions of HIV, behavioral health, and support services outcomes were significantly different between baseline and 12 months (Table 4). We used first difference estimation to assess if characteristics of intervention engagement were associated with HIV outcomes. First difference estimation is an extension of fixed effects regression used to control for all stable characteristics of individuals, observed or unobserved, in longitudinal analyses.^51^

**Table 2.** Baseline Sociodemographic Characteristics (*N* = 66)

Age (mean, range)	27.7 (17–35)
Race, *n* (%)	
Black or African American	66 (100.0)
Ethnicity, *n* (%)	
Latino or Hispanic	2 (3.0)
Sexual orientation, *n* (%)	
Bisexual	12 (18.2)
Gay or homosexual	46 (69.7)
Queer	2 (3.0)
Same gender loving	1 (1.5)
Straight/heterosexual	1 (1.5)
Pansexual	2 (3.0)
Other	2 (3.0)
Education, *n* (%)	
Some high school	7 (10.8)
Graduated high school/GED	13 (20.0)
Some college or technical school	8 (12.3)
Graduated college	6 (9.2)
More than college (graduate school)	1 (1.5)
Annual income, *n* (%)	
≤$5000	22 (33.3)
$5001–$10,000	7 (10.6)
$10,001–$20,000	13 (19.7)
$20,001–$40,000	14 (21.2)
$40,001–$60,000	3 (4.5)
>$60,000	0 (0.0)
Don't know	7 (10.6)
State of residence, *n* (%)	
North Carolina	36 (54.5)
South Carolina	30 (45.5)

GED, General Educational Development.

**Table 3.** Dosage of Intervention (*N* = 66, 992 Encounters)

Frequency of encounters	
Median (range)	10 (1–60)
Encounter duration, % (*n*)	
0–30 min	57.9 (574)
31–60 min	21.7 (215)
60+ min	20.5 (203)
Encounter service category, % (*n*)	
Med case mgmt	33.9 (336)
Non-med case mgmt	6.3 (62)
Mental health	10.7 (106)
Food bank/meal delivery	0.71 (7)
Health ed/risk reduction	4.1 (41)
Housing	7.1 (70)
Medical transport	0.30 (3)
Psychosocial	34.2 (339)
Referral for care	2.8 (28)
Contact type, % (*n*)	
Electronic health record	0.30 (3)
E-mail	2.3 (23)
Group by phone	0.60 (6)
Individual by phone	43.9 (435)
Individual in person	5.0 (50)
Mobile app	0.20 (2)
Text	32.6 (323)
Virtual visit	14.9 (148)
Provider type, % (*n*)	
Behavioral health therapist	11.0 (109)
Health care navigator	89.0 (883)
Provider race same as participant, % (*n*)	
Yes	87.1 (864)

**Table 4.** HIV, Behavioral Health, and Support Services Outcomes (*N* = 66)

	Baseline		6 months		12 months		Fisher
	*n*	%	*n*	%	*N*	%	*p*
Linkage to medical care							
Newly diagnosed clients who attended a routine HIV medical care visit within 3 months of HIV diagnosis	11	91.7					—
Newly diagnosed clients who attended a routine HIV medical care visit in the past 3 months	8	66.7	9	75	8	66.7	0.31
Receipt of HIV medical care							
Clients who had two or more routine HIV medical care visits in the past year	52	78.8	58	87.9	56	84.9	0.43
Retention in HIV medical care							
Clients who had at least one routine HIV medical care visit in the past 12 months, with a second visit at least 90 days after	44	75.9	55	85.9	57	87.7	0.62
Prescribed ART							
Clients prescribed ART in the past 12 months	60	96.8	64	100	64	98.5	1.00
Viral suppression							
Clients with a HIV viral load <200 copies/mL at last HIV viral load test in the past 12 months	51	82.3	58	90.6	59	90.8	0.57
Behavioral health							
Clients screened or assessed for behavioral health (BH) care needs in the past 6 months	45	68.2	59	89.4	47	71.2	0.77
Clients screened positive for BH care needs in the past 6 months	15	33.3	21	35.6	17	36.2	0.20
Clients with positive BH needs referred to BH care in the past 6 months	14	93.3	21	100	14	82.4	0.38
Clients with positive BH needs who have received BH care in the past 6 months	7	50	15	71.4	11	78.6	0.17
Clients with positive BH care needs who have received four or more BH visits in the past 6 months	3	5.3	9	13.8	10	15.4	
Support services							
Clients screened or assessed for SS needs in the past 6 months	58	87.9	59	89.4	52	78.8	
Clients screened positive for SS care needs in past 6 months	47	81	47	79.7	48	92.3	
Clients with positive SS needs referred to SS in the past 6 months	47	100	46	97.9	48	100	
Clients with positive SS needs who have received SS care in the past 6 months	44	93.6	45	97.8	45	93.8	

ART, antiretroviral therapy; BH, behavioral health.

For each outcome, we ran five models: Model 1 examined the association between frequency of encounters with an intervention provider and outcomes; Model 2 examined the association between duration of encounters and outcomes; Model 3 examined the association between category of encounter and outcomes; Model 4 examined the association between type or mode of encounter and outcomes; and Model 5 examined the association between provider type and outcomes. We report odds ratio (OR), 95% confidence interval (CI), and the associated *p* value (<0.05 considered significant). To account for missingness, we used simple imputation. All analyses were conducted using SAS software 9.4.^52^

## Results

### Sample characteristics

As reported in Table 1, mean age was 27.7 years (range: 17–35). The majority of participants identified as gay (69.7%) and 23% had at least some college education. A third of the sample (33.3%) reported a yearly income of $5000 or less. Slightly more than half (54.5%) lived in North Carolina.

### Services utilization, dose, and delivery characteristics

Over the 12-month intervention period, there was a total of 992 encounters between participants and intervention providers. As reported in Table 2, the median number of encounters during the intervention period was 10 (range: 1–60). The majority (57.9%) of encounters lasted between 0 and 30 min and the most common encounter service categories were psychosocial (34.2%), medical case management (33.9%), and mental health (10.7%). Nearly half of encounters were delivered individually by phone (43.9%), with text (32.6%) and virtual visits (14.9%) being the other most common forms of intervention delivery. The vast majority of intervention services were delivered by a health care navigator (89.0%) and by someone of the same race as the participant (87.1%).

### Engagement with mobile app

Client engagement with the study-specific mobile app is described in Table 3. Over the course of the intervention period, the median number of minutes spent in the app was 7.8 (range: 1–698 min) and the median number of logins was 1 (range: 1–29). The median number of forum posts, comments, likes, activity completions, and articles opened, was all 0, although the ranges varied from 0 up to 84. The majority of engagement with the mobile app was by the same five clients.

### HIV, behavioral health, and support services outcomes

We report findings of HIV, behavioral health, and social support outcomes at baseline, 6 months, and 12 months in Table 4. Of the 12 participants who were newly diagnosed, 91.7% (*n* = 11) attended a routine HIV medical care visit within 3 months of their HIV diagnosis. All other HIV outcomes improved from baseline to 12-month follow-up, including receipt of HIV care (78.8–84.9%), retention in HIV care (75.9–87.7%), being prescribed ART (96.8–98.5%), and achieving viral suppression (82.3–90.8%); however, none of these differences were statistically significant.

In general, behavioral health outcomes also improved over the intervention period, although none were statistically significant. The most notable improvement was among clients with a positive behavioral health screening to receive behavioral health care in the previous 6 months (50.0–78.6%; *p* = 0.17). We found mixed results among support services outcomes. Over the 12 months, the proportion of clients screened for support services needs in the previous 6 months fell from 87.9% at baseline to 78.8% at 12 months. All other social support outcomes saw positive trends.

### Multi-variable analyses

In multi-variable analyses (Table 5), we found a significant association between encounter service category and being prescribed ART (*p* < 0.01) and achieving viral suppression (*p* = 0.04). Participants with more encounters categorized as food bank or meal delivery were more likely to report being prescribed ART (OR: 41.65; 95% CI: 2.72–637.74, *p* = 0.01) compared with clients who reported more psychosocial encounters.

**Table 5.** Multi-Variable Results of Intervention Dosage on HIV outcomes

		Receipt of HIV medical care			Retention in HIV medical care			Prescribed ART			Viral suppression		
		OR	95% CI	*p*	OR	95% CI	*p*	OR	95% CI	*p*	OR	95% CI	*p*
Model 1: Frequency of encounters				0.40			0.17			0.79			0.24
	Frequency of encounters	1.02	0.98–1.05	0.38	1.02	0.99–1.06	0.18	0.99	0.94–1.05	0.79	1.02	0.99–1.06	0.24
Model 2: Encounter duration				0.53			0.65			0.52			0.41
	31–60 min	1.06	0.96–1.17	0.25	1.04	0.94–1.14	0.48	0.93	0.81–1.07	0.32	1.07	0.97–1.18	0.19
	60+ min	0.99	0.85–1.15	0.86	1.03	0.89–1.18	0.72	0.96	0.77–1.20	0.74	0.97	0.84–1.13	0.73
Model 3: Encounter service category				0.27			0.14			**<0.01**			**0.04**
	Medical case mgmt	1.11	0.93–1.31	0.20	1.15	0.99–1.34	0.07	1.21	0.95–1.55	0.12	1.09	0.90–1.32	0.40
	Non-med case mgmt	0.96	0.61–1.52	0.86	0.97	0.61–1.55	0.90	1.59	0.84–3.04	0.16	1.36	0.88–2.09	0.16
	Mental health	1.10	0.95–1.27	0.20	1.07	0.94–1.22	0.30	1.03	0.71–1.51	0.87	1.09	0.96–1.25	0.19
	Food bank/meal delivery	1.53	0.32–7.37	0.59	2.67	0.57–12.60	0.21	41.65	2.72–637.74	**0.01**	2.87	0.58–14.2	0.20
	Health ed/risk reduction	0.72	0.39–1.31	0.28	1.38	0.79–2.40	0.26	1.06	0.36–3.07	0.92	0.73	0.40–1.36	0.33
	Housing	0.83	0.64–1.09	0.17	0.93	0.71–1.20	0.56	1.13	0.77–1.68	0.53	0.89	0.67–1.18	0.42
	Medical transport	0.85	0.09–8.10	0.89	0.08	0.004–1.42	0.09	0.004	<0.001–1.06	**0.05**			
	Referral for care	0.77	0.35–1.70	0.51	0.59	0.23–1.50	0.27	0.02	<0.001–0.42	**0.01**	0.39	0.18–0.84	**0.02**
Model 4: Contact type				0.34			0.60			0.12			0.40
	Electronic health record	0.93	0.03–30.31	0.97	0.77	0.03–21.65	0.88	0.10	<0.001–268.35	0.57	0.33	0.01–8.28	0.50
	E-mail	0.45	0.20–1.02	0.06	0.81	0.37–1.76	0.59	1.40	0.51–3.86	0.52	0.58	0.26–1.30	0.18
	Group by phone	1.50	0.17–13.45	0.72	0.87	0.10–7.30	0.89	0.13	0.002–10.05	0.36	2.73	0.35–21.48	0.34
	Individual in person	1.01	0.73–1.41	0.93	1.23	0.86–1.78	0.26	1.19	0.73–1.95	0.50	1.13	0.77–1.65	0.54
	Mobile app	0.65	0.02–18.86	0.80	2.76	0.16–47.16	0.48	0.39	0.001–152.15	0.76	0.50	0.02–12.96	0.68
	Text	1.18	1.02–1.35	**0.02**	1.08	0.95–1.23	0.24	1.08	0.91–1.27	0.40	1.12	0.98–1.28	0.10
	Virtual visit	1.02	0.92–1.12	0.77	1.04	0.94–1.14	0.45	0.93	0.79–1.12	0.47	1.03	0.93–1.13	0.58
Model 5: Provider type				**0.03**			0.11			0.87			0.07
	BH therapist	1.13	1.00–1.28	**0.05**	1.09	0.97–1.22	0.14	0.99	0.84–1.16	0.87	1.10	0.98–1.23	0.10

CI, confidence interval; OR, odds ratio.

*p*-Values ≤ 0.05 are in bold.

However, clients with more referral to care encounters were less likely to have been prescribed ART (OR: 0.02; 95% CI: <0.001–0.42; *p* = 0.01) and be virally suppressed (OR: 0.39; 95% CI: 0.18–0.84; *p* = 0.02). We also found that provider type was significantly associated with receipt of HIV medical care (*p* = 0.03). Clients who reported more intervention services delivered by a behavioral health therapist were more likely to receive HIV medical care (OR: 1.13; 95% CI: 1.00–1.28; *p* = 0.05) compared with those who received services from a health care navigator. Frequency of encounters, duration of encounters, and contact type were not significantly associated with HIV outcomes.

## Discussion

Overall, we found positive trends across all HIV care continuum outcomes among clients who participated in our multi-component behavioral intervention. Although none of these changes were statistically significant, they suggest that an integrated approach to HIV and behavioral health services may help BMSM living with HIV overcome structural and social barriers to HIV care. This is also reflected in the increase in proportion of clients with a positive behavioral health screening who received behavioral health care in the previous 6 months (from 50.0% to 78.6%).

Our mixed findings from multi-variable analyses highlight the complex role that structural barriers play in the HIV care continuum among BMSM living with HIV. Evidence shows that a many different structural and other factors can influence outcomes at each stage of the HIV care continuum, including poverty, lack of health insurance, homelessness, food insecurity, substance use, mental health problems.^53^ For example, our finding that clients with more food bank or meal delivery encounters were more likely to be prescribed ART suggest that providing structural support to individuals struggling to have their basic needs met may also improve their HIV outcomes.

Addressing structural barriers has become even more critical for BMSM living with HIV during the COVID-19 pandemic as other studies have found that Black lesbian, gay, bisexual, transgender, queer (LGBTQ) individuals were more severely affected by COVID-19 than non-LGBT and LGBT of other races/ethnicities in terms of serious financial problems and their ability to get timely medical care.^54^ Research also finds that LGBTQ people, particularly LGBTQ people of color and those with disabilities, are more likely than their peers to experience food insecurity. Data from several nationally representative surveys found that more than one in four (27%) LGBTQ adults experienced a time in the last year when they did not have enough money to feed themselves or their families, compared with 17% of non-LGBTQ adults.

Within the community, 42% of Black LGBTQ people, 33% of Hispanic LGBTQ people, and 31% of LBT women reported not having enough money for food in the prior year.^55,56^ As our findings suggest, providing integrated support for BMSM living with HIV, such as food banks or meal deliveries, is crucial for ending the HIV epidemic.

Even with the additional support provided by HCN and behavioral health therapists, some clients required additional support beyond the scope of the intervention and were referred to outside care, such as assistance with obtaining prescription medications. Our findings that these clients were less likely to be prescribed ART and virally suppressed may indicate that these individuals experienced additional barriers that were not addressed by the intervention and, therefore, were more likely to experience worse HIV outcomes. Similar results were reported by a health navigator intervention among MSM in Guatemala, which also found that clients who required more support from navigators and external sources experienced worse HIV and mental health outcomes.^57^ Additional research is needed to determine if and how integrated HIV and behavioral health services can ensure that individuals who are referred to external services receive the support they need to achieve and maintain viral suppression.

Although our findings suggest that a multi-pronged approach to improving HIV and behavioral health outcomes among BMSM can be successful, the low engagement with the STYLE 2.0 healthMpowerment app may suggest that there is a limit in the amount and types of services that can be offered in multi-component behavioral interventions. We do not believe this is a reflection on the utility of the app, which has been successful in other interventions,^48,58,59^ but that when offered alongside client-centered human interaction with a navigator or behavioral therapist, is less prioritized by clients.

### Limitations

There are several limitations to our study. Because of the nature of our study design and lack of a control group, we are unable to assess causation. However, we used an approach that allowed us to approximate change in HIV outcomes over the same period clients were exposed to the intervention, controlling for all time-invariant variables. The COVID-19 pandemic impacted our ability to recruit participants in-person in clinics as well as through community partner events, limiting our sample size. This likely affected our ability to see significant associations and contributed to the wide CIs reported.

However, the STYLE 2.0 intervention originally consisted of both in-person and virtual programming, therefore transitioning to all virtual activities during the COVID-19 pandemic was not a large deviation from the original planned intervention. In addition, as other studies conducted during the pandemic have discussed,^60^ COVID-19 may have resulted in lower engagement with the intervention that may have muted its effectiveness.

To reduce the HIV disparities experienced by Black MSM in the US South, multi-component behavioral interventions that address structural barriers to HIV care are critical. The STYLE 2.0 intervention provides a successful foundation from which future interventions that target Black MSM living with HIV may be designed.
